# Genotype–phenotype analysis in 333 individuals with heterozygous variants in *NFKB1*

**DOI:** 10.70962/jhi.20260076

**Published:** 2026-10-19

**Authors:** Sophia Marie Armbruster, Andres Caballero-Oteyza, Oliver Bartsch, Tiziana Lorenzini, Hassan Abolhassani, Samantha Chan, Luis Ignacio González Granado, Victor Garcia-Bustos, Marta Dafne Cabañero Navalon, Nazanin Fathi, Nima Rezaei, Mathieu Fusaro, Roger Colobran, Kaan Boztug, Michele Proietti, Bodo Grimbacher

**Affiliations:** 1 Faculty of Medicine, Clinic of Rheumatology and Clinical Immunology, Center for Chronic Immunodeficiency, Medical Center, Albert-Ludwigs-University of Freiburg, Freiburg, Germany; 2 Faculty of Medicine, Institute for Immunodeficiency, Center for Chronic Immunodeficiency, Medical Center, Albert-Ludwigs-University of Freiburg, Freiburg, Germany; 3 German Center for Infection Research, Satellite Center Freiburg, Freiburg, Germany; 4 Centre for Integrative Biological Signaling Studies, Albert-Ludwigs University, Freiburg, Germany; 5 RESIST – Cluster of Excellence 2155 to Hanover Medical School, Satellite Center Freiburg, Freiburg, Germany; 6 Department of Human Genetics, Medical Care Center, University Medical Center of the Johannes Gutenberg University Mainz, Mainz, Germany; 7 Division of Immunology and Children’s Research Center, University Children’s Hospital Zürich, Zürich, Switzerland; 8 Division of Immunology, Department of Medical Biochemistry and Biophysics, Karolinska Institute, Stockholm, Sweden; 9 Department of Clinical Immunology & Allergy, Royal Melbourne Hospital, Melbourne, Australia; 10 Primary Immunodeficiencies Unit, Department of Pediatrics, Hospital 12 Octubre, Research Institute University Hospital 12 de Octubre Complutense School of Medicine, Madrid, Spain; 11 Primary Immunodeficiencies Unit, Department of Internal Medicine, University and Polytechnic Hospital La Fe, Valencia, Spain; 12 Severe Infection Research Group, Health Research Institute La Fe, Valencia, Spain; 13 Research Group of Chronic Diseases and HIV Infection, Health Research Institute La Fe, Valencia, Spain; 14 Research Center for Immunodeficiencies, Pediatrics Center of Excellence, Children’s Medical Center, Tehran University of Medical Sciences, Tehran, Iran; 15 Immunology Department Laboratory, Referral Medical Biology Laboratory, Toulouse University Hospital Center, Toulouse, France; 16 INFINITY - Toulouse Institute for Infectious and Inflammatory Diseases, INSERM, U1291, CNRS U5051, University Toulouse III, Toulouse, France; 17 Translational Immunology Research Group, Immunology Division, Department of Cell Biology, Physiology and Immunology, Vall D’Hebron Research Institute, Vall D’Hebron University Hospital, Autonomous University of Barcelona, Barcelona, Spain; 18 Clinic for Pediatric Immunology and Rheumatology, University of Bonn, University Hospital Bonn, Bonn, Germany; 19 St. Anna Children’s Cancer Research Institute, Vienna, Austria; 20 Department of Pediatrics and Adolescent Medicine, Medical University of Vienna, Vienna, Austria; 21 CeMM Research Center for Molecular Medicine of the Austrian Academy of Sciences, Vienna, Austria

## Abstract

Heterozygous germline variants in *NFKB1* can be classified into variants with “known protein defect” and “unknown protein effect.” However, beyond this, *NFKB1* variants may be additionally categorized into at least eight distinct variant classes. We conducted a literature review of 67 papers reporting on *NFKB1* variants and included four additional individuals, carrying previously unreported *NFKB1* variants. We identified 333 individuals with unique variants in *NFKB1*. Our compiled data revealed that there is no sex predilection, the penetrance—including all heterozygous carriers—was 78%, and the mean age of onset for the “known protein defect cohort” was 21.2 years. Individuals harboring variants with a known protein defect demonstrated a higher frequency of malignancies and autoimmune disorders. However, these individuals were less frequently classified as affected than individuals with variants with unknown protein defect. Carriers of whole *NFKB1* gene deletions had a significantly higher penetrance to being designated as “affected,” but beyond this, the clinical manifestations did not differ between variant classes.

## Teaser

Our literature review included all currently published patients with NFKB1 variants. Variants were divided into subcohorts and classified into distinct variant classes. Correlative analyses were performed to search for genotype–phenotype correlations.

## Introduction

NF-κB signaling is a complex network of various interacting intracellular messenger molecules, leading to feed-forward and inhibitory/reverse signaling cues (1). The NF-κB signaling system consists of seven core proteins: the cytoplasmic precursors p105 (encoded by *NFKB1*) and p100 (encoded by *NFKB2*), which are processed into the transcription factor subunits p50 and p52, respectively, and their dimerization partners RelA (p65), Rel-B, and c-Rel. The canonical NF-κB1–dependent signaling pathway is responsible for cell activation and inflammation, activating proinflammatory cytokines and chemokines and anti-apoptotic factors, whereas the noncanonical NF-κB2–dependent pathway is preferentially involved in cell-lineage commitment and cellular differentiation processes. The p105 precursor (comprising 969 amino acids—ENSP00000226574/NP_003989.2) is converted into the mature NF-κB1 transcription factor subunit p50 (comprising the N-terminal 433 amino acids of p105) by proteasomal processing.

A heterodimeric NF-κB transcriptional activator consists of one NF-κB subunit and one of the Rel proteins, which provides the transcriptional transactivation domain (TAD). The most prevalent canonical NF-κB transcriptional activators are the p50/RelA dimers, while p50:p50 homodimers are transcriptional repressors as they lack a TAD. All NF-κB and Rel proteins share the N-terminal Rel homology domain (RHD), responsible for dimerization, DNA binding, and inhibitory NF-κB protein (IκB) interaction. Preformed NF-κB transcription factors are retained within the cytoplasm by inhibitory proteins (IκBs, which also include the precursors). Upon pathway activation, the IκBs undergo decay and the released NF-κB dimers translocate to the nucleus to regulate their target genes.

Monoallelic impairments in this vulnerable system in humans may phenotypically result in immunodeficiency, autoinflammation, and autoimmune conditions such as cytopenia, enteropathy, and autoimmune dermatologic disease, but malignant diseases are also frequently present (2, 3). Most of the hitherto identified *NFKB1* variants comprise loss-of-function severe protein truncations and deleterious single amino acid changes within the RHD, which both cause protein loss and insufficiency of p105 and p50 in affected carriers (3, 4, 5). Notably, pathogenic *NFKB1* variants have been reported to be the most frequent monogenic cause of common variable immunodeficiency (CVID) (6). So far, the highly heterogeneous manifestations and the incomplete penetrance associated with genetic variants in *NFKB1* have prohibited the correlation of the genotypes and their associated disease phenotypes (3). This might be due to the variety of pivotal functions executed through NF-κB signaling within the immune system. Yet, the phenotypic heterogeneity might be—at least in part—due to genotypic heterogeneity, which causes a diversity of protein defects, and which might disturb NF-κB signaling at multiple levels.

A previous systematic review from Fathi et al. comprehensively catalogued all previously reported variants in *NFKB1* and *NFKB2* up to February 2024 and compared the *NFKB1* phenotype with the one of *NFKB2* (7). In contrast, our study asked the question whether there is a phenotypic difference between *NFKB1* variant carriers of various variant classes, leading to different protein defects. We compiled all published carriers of *NFKB1* variants (up to December 2024) and confirmed the high heterogeneity and incomplete penetrance of their associated phenotypes. We then tested whether the phenotypic heterogeneity is reduced when subcohorts representing uniform protein defects are analyzed separately.

**Figure 1.**
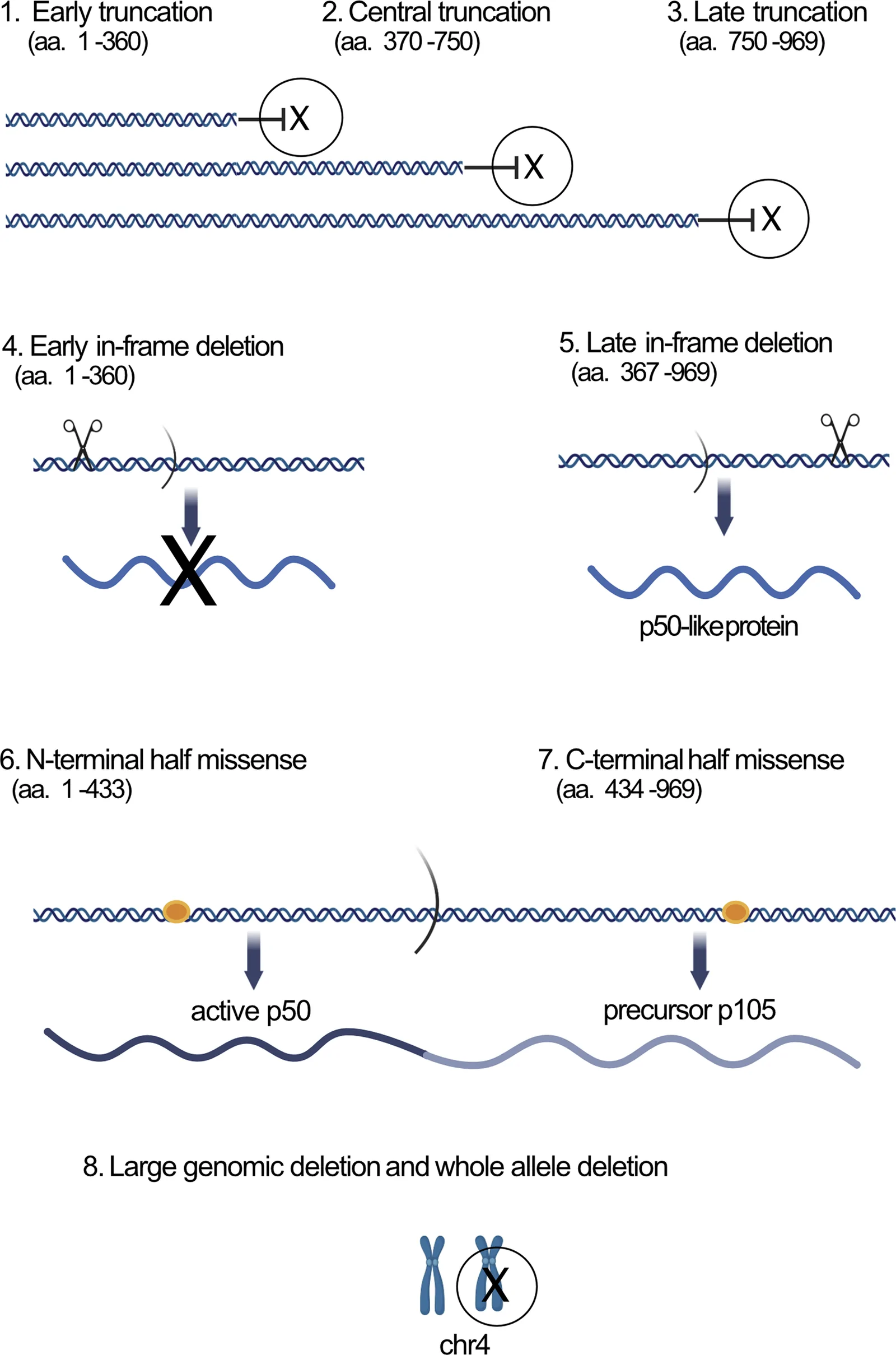
**Domains of the NF-κB1 protein with protein defect groups.** Variant classes: (1) early truncations, which result from nonsense or frameshift variants, typically on the N-terminal side relative to the NLS, and cause loss of p105 and p50 expression; (2) central truncations, which predict the immediate expression of p50-like proteins instead of being generated from their p105 precursors (precursor skipping); (3) late truncations, which affect only the C-terminal domains of p105; (4) early in-frame deletions, mainly resulting from splicing errors, which can cause expression of mutant precursors which, however, undergo accelerated decay instead of being processed to p50; (5) late in-frame deletions, which are particularly rare but could be converted to p50, if processing is enabled; (6) missense variants residing within the N-terminal p50 half, including the frequent protein-decaying variants and sporadic hypomorphic and loss-of-function variants with normal protein stability; (7) missense variants residing within the C-terminal half of p105 and most likely affecting only p105-specific functions; and (8) large genomic deletions involving the entire NFKB1 or parts thereof. Figure not to scale, schematic representation. aa, amino acid; NLS, nuclear localization sequence; chr4, chromosome 4.

*NFKB1* variants that have been tested and confirmed to be pathogenic, or *NFKB1* variants that have been predicted to be pathogenic or likely pathogenic, can be separated as (1) variants with a “known protein defect” and (2) variants with an unknown effect on protein function on p105 or p50, respectively. Hence, the “known protein defect cohort” included nonsense variants, missense variants with functional evidence from in vitro tests, and splice site variants with evidence for alternative splicing. Beyond this simple binary grouping, variants may additionally be categorized into at least eight distinct “variant classes” (3, 5, 6) depending on the predicted effect on the encoded protein(s). These include (early/central/late) protein truncating variants, internal in-frame deletions with/without a resulting p50-like protein and missense variants occurring in the N-terminal p50-half or the C-terminal p105-specific half, and large genomic deletions including *NFKB1* (Fig. 1). While the outcome of short truncating variants appears to be solved (they all lead to *NFKB1* haploinsufficiency), the impact of central and late truncating variants, if they result in protein expression, is largely unclear. Only the damaging effects (such as protein-decaying, loss of DNA-binding, and sporadically hypomorphic effects) can be confirmed experimentally in vitro (3, 4, 8, 9) with some accuracy, while subtle defects and the role of variants assumed to predominantly affect p105-specific functions (e.g., late truncating variants or C-terminal missense variants) remain obscure. Since currently available functional tests do not cover all protein functions (e.g., protein–protein interactions or posttranslational modifications of the protein), the role of the particularly rare variants with “neutral” test (isomorphic missense variants) also remains uncertain. Moreover, beyond a high allele frequency in the population, there are no methods available to confirm benignity.

We aimed at identifying common disease features coinciding with specific variant classes. Unexpectedly, no clear correlation of the phenotype with the underlying genotype was identified, although the biochemical defects in each group were clearly distinct. Because a single common disease mechanism for all variant types appears conceivable, we discuss the enigmatic causalities of the variable penetrance and expressivity of *NFKB1*-related disease, which occur even among carriers of the same variant and within the same family.

## Results

The *NFKB1*-associated disease phenotype is highly heterogeneous with variable expressivity and incomplete penetrance. In this study, we conducted a comprehensive literature review to investigate whether this remarkable heterogeneity is at least partially due to the variety of the underlying genetic lesions.

### Cohort characteristics

**Figure 2.**
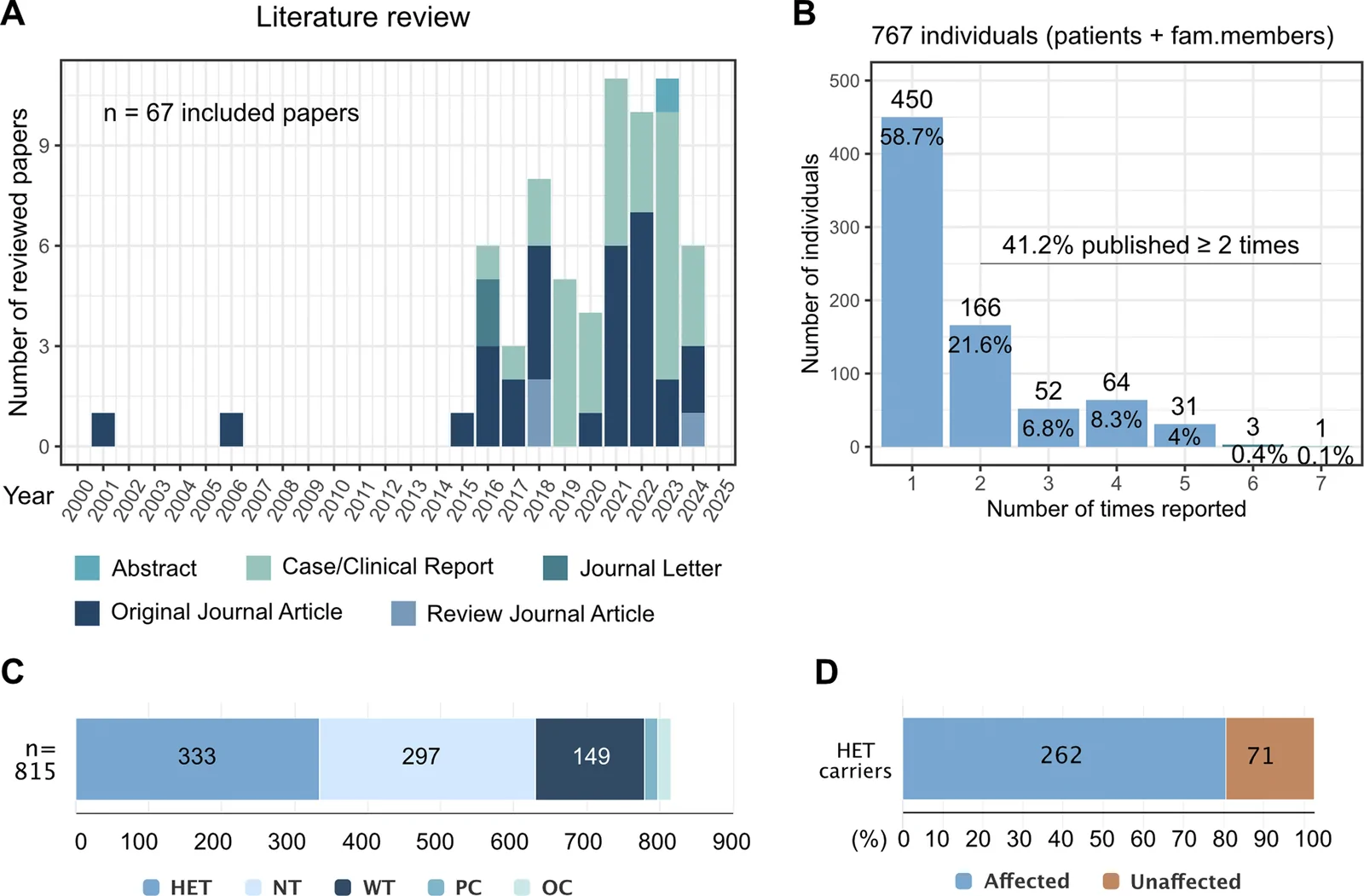
**Cohort characteristics and literature review. (A)** 67 case reports and original articles, specialist or scientific articles, review articles, and one abstract were evaluated. **(B)** Our retrospective literature research revealed 767 individuals, of whom more than 40% were reported twice or more. **(C)** Cohort characteristics of the total cohort. Of all identified 815 individuals, 333 were confirmed heterozygous NFKB1 variant carriers, 297 relatives were not genetically evaluated, 149 relatives were confirmed to be wild-type, 17 were obligate heterozygous carriers, and 19 were presumed carriers. **(D)** Of the 333 NFKB1 variant carriers, 262 (79%) were reported as clinically affected and 71 (21%) were reported unaffected at the time of publication. Fam., family; HET, heterozygous; NT, not tested; WT, wild type; PC, presumed carrier; OC, obligate carrier.

The thorough inspection of the 67 relevant papers (Fig. 2 A) and contact with the corresponding authors/physicians allowed us to identify a total of 815 individuals (Table S1). These include index cases, and affected and unaffected family members, who were either heterozygous, wild-type, or had not been genetically tested for *NFKB1*. Included are also previously unreported patients and their relatives under medical attendance at the Center for Chronic Immunodeficiency (CCI) in Freiburg (shown in blue in Table S2). We found at least 41.2% of the previously reported individuals to be reported more than once, and actually up to seven times in one case (Fig. 2 B). If we consider only *NFKB1* variant carriers, the percentage of subjects reported more than once is 48.4% (not shown). Because the linkage region for this condition was published earlier (10, 11), clinical reports on one large family (AA) were released before the first description of *NFKB1* variants as a monogenic cause of inborn errors of immunity (5).

Of all identified 815 individuals, 333 were confirmed heterozygous *NFKB1* variant carriers (Fig. 2 C and Table 1). Of the 333 confirmed carriers, 262 (78.7%) were clinically affected and 71 (21.3%) were reportedly unaffected at the last time of evaluation (Fig. 2 D). Within this cohort, 90 cases were described as sporadic. We identified 88 familial cases, drew their pedigrees (Data S1) for in-depth family analysis, and thereby identified 17 obligate and 19 presumed *NFKB1* variant carriers. We decided to consider 350 individuals as the “total *NFKB1* cohort” (333 heterozygous and 17 obligate carriers). They carried a total of 157 distinct variants in *NFKB1* (Table S3).

**Table 1.** Cohort shown according to variant classes and zygosity

Variant type/deduced protein defect	HET	OC	PC	WT	Not tested
(1) Early truncations	118	2	5	63	​
(2) Central truncations (precursor-skipping variants)	54	8	7	20	​
(3) Late truncations	7	—	1	7	​
(4) Early in-frame deletions	40	5	—	31	​
(5) Late in-frame deletions	1	—	—	—	​
(6) N-terminal half missense	86	2	5	22	​
(7) C-terminal half missense	10	—	1	3	​
(8) Large genomic deletions and whole allele deletions	17	—	—	3	​
Unknown	—	—	—	—	297
Individuals in total	333	17	19	149	297

OC, obligate carrier; PC, presumed carrier; HET, heterozygous carrier; WT, wild type.

The *NFKB1* cohort comprised the following nationalities and/or ethnicities: German (106), Finnish (100), Dutch (94), Caucasian (not specified) (163), Turkish (39), Chinese (26), Iranian (26), Spanish (16), Italian (15), Israeli (9), Russian (8), American (7), British (7), Japanese (7), European/American (5), Danish (5), Filipino (4), Taiwanese (4), Australian (3), Ecuadorian (3), Asian (2), Indian (2), Brazilian (1), Egyptian (1), Portuguese (1), unknown (161).

### Division of the total *NFKB1* cohort in two subcohorts

**Figure 3.**
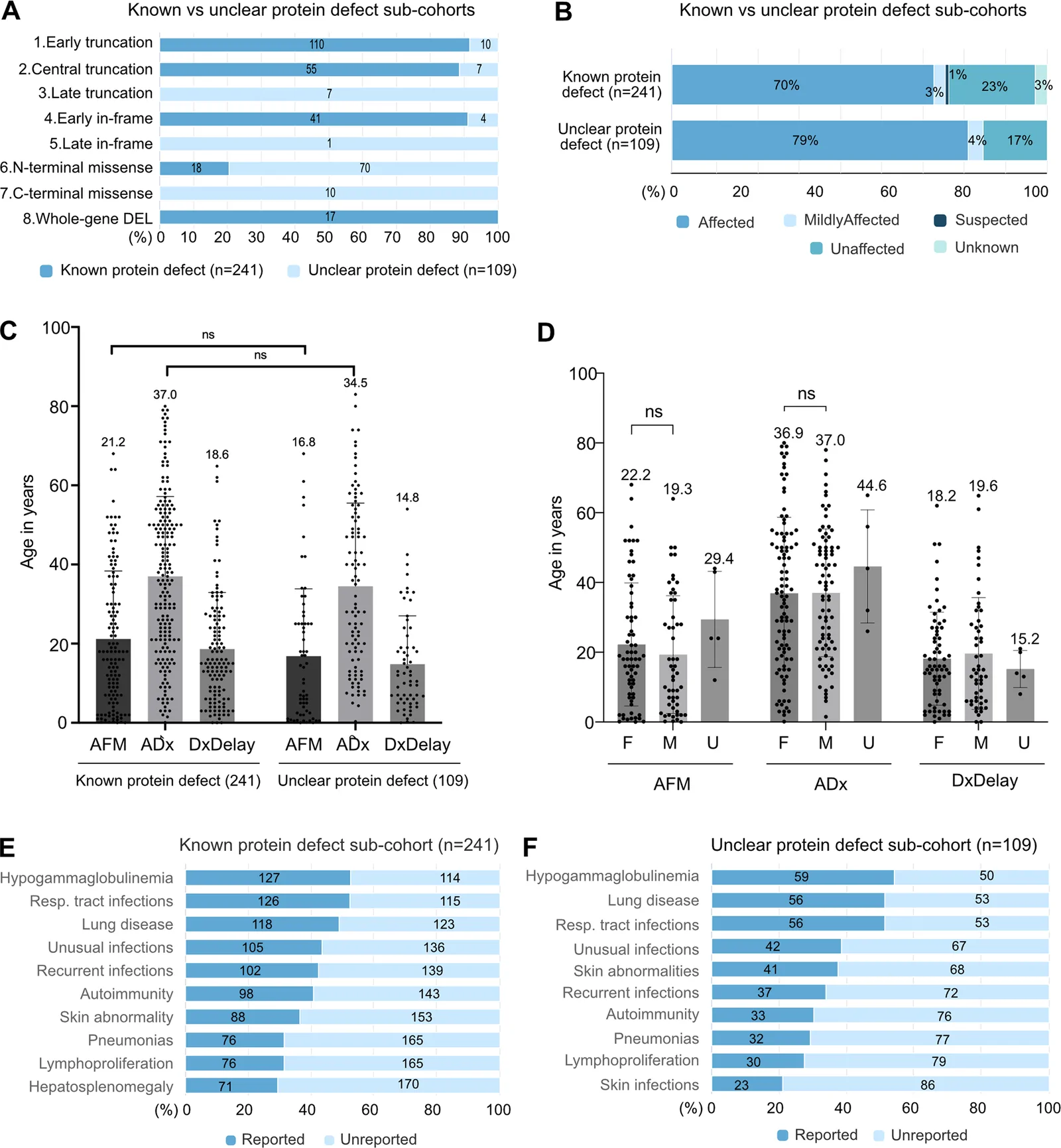
**Comparison of the clinical characteristics between the sub-cohorts. (A)** Number of individuals per variant class within the known protein defect cohort, *n*= 241 (dark blue), and the remaining protein defect unclear cohort, *n*= 109 (lighter blue). **(B)** Disease status for the known protein defect cohort, *n* = 241, and the protein defect unclear cohort, *n* = 109. **(C)** Comparison of AFM (dark gray), ADx (light gray), and DxDelay (time between appearance of the first symptoms and genetic diagnosis, middle gray) between the known protein defect cohort, *n* = 241, and the protein defect unclear cohort, *n* = 109. AFM for the known protein defect cohort, *n* = 241, was 21.2 years, and age at diagnosis was 37 years. The average duration between symptom manifestation and genetic diagnosis was 18.5 years. In comparison, the AFM for the protein defect unclear cohort, *n* = 109, was 16.8 years, the age at genetic diagnosis was 34.5 years, and the average duration between symptom manifestation and the genetic diagnosis was 14.8 years. **(D)** Comparison of AFM, ADx, and DxDelay (time between appearance of the first symptoms and genetic diagnosis) between sex categories in the known protein defect cohort, *n* = 241. The mean AFM was observed to be 22.2 years for female (F) patients, 19.3 years for male (M) patients, and 29.4 years for patients with unknown (U) sex. The mean age at genetic diagnosis was 39.9 years for female individuals, 37 years for male individuals, and 44.6 years for patients with unknown sex. The time between the initial manifestation and the confirmation of the genetic diagnosis was longest for men with 19.6 years, and shortest for patients of unknown sex with 15 years. **(E)** 10 most often reported clinterms for the known protein defect cohort, *n* = 241. **(F)** 10 most often reported clinterms for the protein defect unclear cohort, *n* = 109. AFM, age at first manifestation; ADx, age at genetic diagnosis; DxDelay, diagnostic delay.

Preliminary analyses of the entire cohort confirmed the previous observation of highly heterogeneous phenotypes among the affected *NFKB1* variant carriers. We therefore asked whether this heterogeneity is at least partially due to carriers of variants with known protein defects or variants with unpredictable protein defects. We evaluated all 157 reported monoallelic *NFKB1* variants and concluded that 93 of the 157 variants with confirmed or assumed loss of expression can be classified in a homogeneous “variant with known protein defect” subcohort (Table S3). These 93 variants with known protein defects were present in 241 individuals out of the 350 individuals with *NFKB1* variants (Fig. 3 A).

Specifically, among the 120 individuals carrying “early truncations” 110 belong to the “variant with known protein defect cohort.” The second largest group are “central truncations.” Of these 62 individuals, 55 subjects harbored variants of the known protein defect cohort. The “late truncation” class (affecting only the C-terminal domains of p105) included only seven individuals, but the effects of these variants on the p105/p50 protein functions are all unknown, so these seven subjects were included in the “variants with unclear protein defect cohort.” In the “early in-frame deletions” class, there were 45 subjects, 41 of whom carried variants which were confirmed in the known protein defect cohort. The only carrier of a “late in-frame deletion” was classified within the “variants with unclear protein defect” cohort. 88 individuals had a variant classified as “N-terminal half missense.” Out of these 88, 18 individuals were grouped into the known protein defect cohort. Finally, all 15 individuals with “large genomic deletions” including *NFKB1* and the two brothers with the 5′UTR deletion were included in the known protein defect cohort. The latter two patients showed severely decreased *NFKB1* mRNA expression levels and decreased p50 and p105 expression in T cells.

#### Comparison of the known protein defect cohort with the “unclear protein defect cohort”

Of the 241 individuals of the known protein defect cohort, 74% were considered clinically affected (70% clearly affected and 4% mildly affected) and 23% were clinically asymptomatic at the time of evaluation. 3% of participants were phenotypically uncharacterized. Therefore, they were excluded from statistical analysis. The remaining variants with unclear protein defect cohort comprised 109 individuals with a heterogeneous assortment of variants with unknown/unpredictable protein defects. Among this variants with unclear protein defect cohort, 83% were affected and 17% were unaffected (Fig. 3 B). Therefore, penetrance was slightly but significantly lower (P = 0.0417) in the known protein defect cohort.

Next, we calculated the mean age at first manifestation (AFM), mean age at genetic diagnosis, and the diagnostic delay, defined as the interval between symptom onset and definitive genetic diagnosis. AFM for the known protein defect cohort was 21.2 years, and age at diagnosis was 37 years. The average duration between symptom manifestation and the genetic diagnosis was 18.5 years. In comparison, the AFM for the 109 individuals harboring variants with unclear protein defect was 16.8 years, the age at genetic diagnosis was 34.5 years, and the diagnostic delay was 14.8 years (Fig. 3 C). There was no statistically significant difference between the two subcohorts.

We also did not observe a significant sex bias (P > 0.05 for all comparisons) regarding AFM (females: 22.2 years; males: 19.3 years) and age at genetic diagnosis (females: 40 years; males: 37 years), although the known protein defect cohort had 50% females and 46% males (4% remained unspecified due to missing information), while the “protein defect unclear cohort” had 40% females and 60% males (Fig. 3 D).

#### Phenotypical comparison of the subcohorts

**Figure S1.**
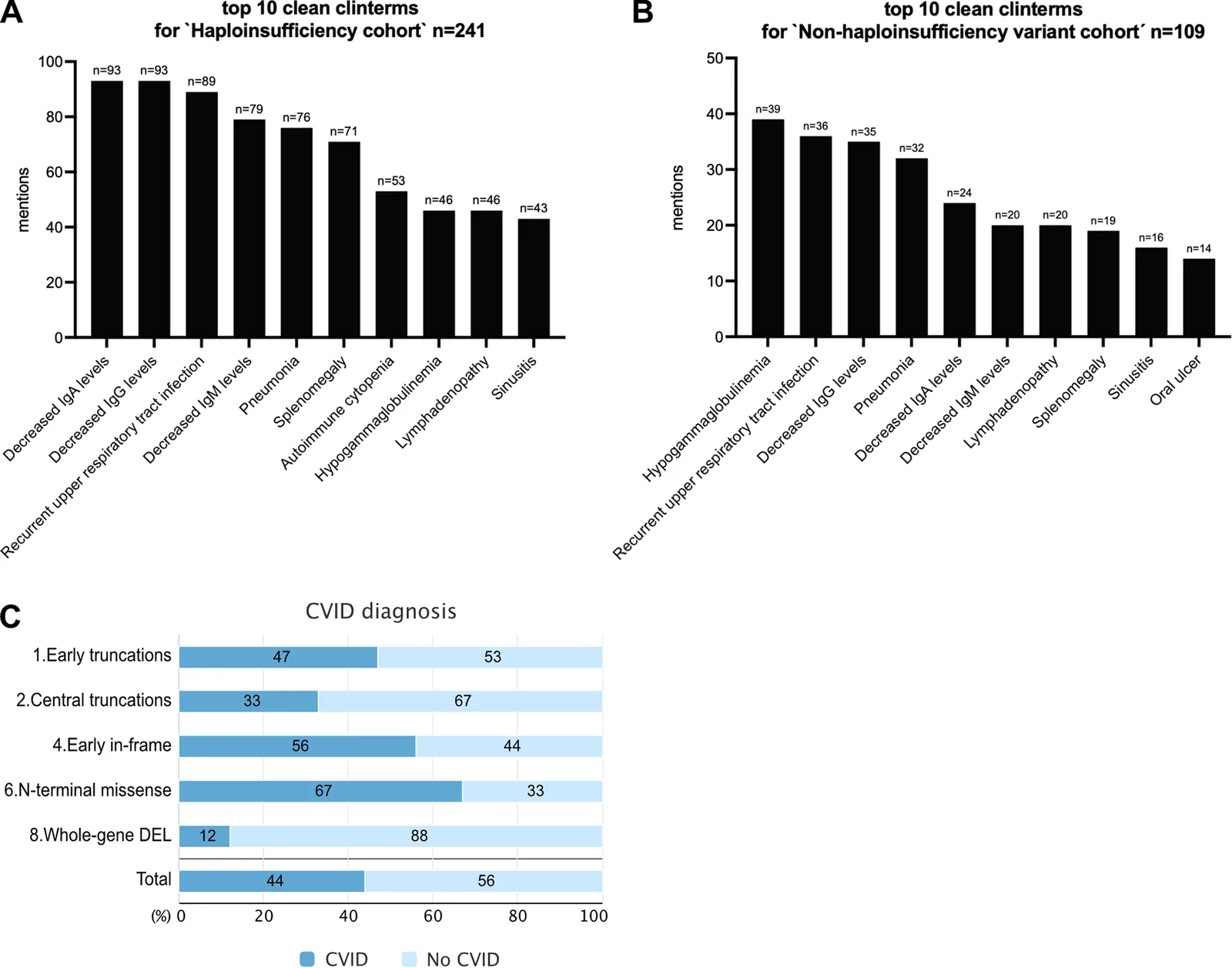
**Additional clinical characteristics. (A)** 10 most often reported simple clinterms for the known protein defect cohort, *n* = 241. **(B)** 10 most often reported simple clinterms for the protein defect unclear cohort, *n* = 109. **(C)** Prevalence of CVID diagnosis for each variant class. The analysis of the prevalence of CVID among different variant classes revealed statistically significant differences in the frequency of occurrence (significance level of P < 0.05), followed by post hoc pairwise comparisons with the following specific findings: class 1: early truncation vs. class 8: large genomic deletions and whole allele deletion (P = 0.0072); class 2: central truncation vs. class 4: early in-frame deletions (P = 0.0364); class 2: central truncation vs. class 6: N-terminal half missense mutations (P = 0.0144); class 4: early in-frame deletions vs. class 8: large genomic deletions and whole allele deletion (P = 0.0029); and class 6: N-terminal half missense mutations vs. class: 8 large genomic deletions and whole allele deletion (P = 0.0016). These findings suggest that individuals with NFKB1 variants from classes 4 and 6 are more likely to be diagnosed with CVID than individuals with variants that cause other protein defects. One of the most frequent manifestations of heterozygous NFKB1 variants is hypogammaglobulinemia, most often manifesting as CVID. Vice versa, variants in NFKB1 are the most frequent monogenic cause of CVID, long known as a very heterogeneous disease, hence the “variable” in its name. Identifying NFKB1 variants in any given CVID patient is of paramount importance, as it not only enables genetic counseling for family members (shortening the diagnostic delay) and family planning, but most importantly offers personalized treatment options.

We next determined the 10 most frequent clinical features within the total *NFKB1* cohort by analyzing the ungrouped clinical term (clinterm) frequency: (1) decreased IgG levels, (2) recurrent upper respiratory tract infection, (3) decreased IgA levels, (4) pneumonia, (5) decreased IgM levels, (6) splenomegaly, (7) hypogammaglobulinemia, (8) lymphadenopathy, (9) autoimmune cytopenia, and (10) (recurrent) sinusitis. While the known protein defect cohort had the same top 10 clinterms, yet with different frequencies, we found the protein defect unclear cohort to exhibit a distinct pattern, notably with oral ulcers (Fig. S1, A and B).

Using clinterm groups (gr), the 10 most frequent conditions for both the known protein defect cohort and the protein defect unclear cohort were identical but had different frequencies: (1) hypogammaglobulinemia gr, (2) (unusual) respiratory tract infection gr, (3) lung disease gr, (4) unusual infection gr, (5) recurrent infections gr, (6) autoimmunity gr, (7) skin abnormality gr, (8) pneumonia gr, (9) abnormal lymphocyte proliferation (lymphoproliferation) gr, and (10) hepatosplenomegaly gr (Fig. 3, E and F). We found the known protein defect cohort to be different from the protein defect unclear cohort with regard to their frequency, while the disease groups themselves did not differ. Particularly, respiratory tract infections retained their high priority. In both cohorts, upper respiratory tract infections remain the most often mentioned clinterm in the group of (unusual) respiratory tract infection. Hypogammaglobulinemia and especially decreased IgG levels thus emerged as the most common complication in *NFKB1*-insufficient patients. Hypogammaglobulinemia results in an increased susceptibility to infections.

Considering the comparison of selected disease features between the subcohorts, individuals carrying variants causing known protein defects exhibited a notably higher frequency of both malignancies (38 vs. 25%; P = 0.0268) and autoimmune disorders (10 vs. 3%; P = 0.0202), compared with the protein defect unclear cohort (Table 2). Apart from these differences, the remaining disease features showed no statistically significant variations between the two subcohorts.

**Table 2.** Crosstable for disease features for the known protein defect cohort and the protein defect unclear cohort

Disease manifestation	Known protein defect cohort		Protein defect unclear cohort	
	# of patients	%	# of patients	%
1. Autoimmunity	91	38	27	25
2. Lymphoproliferation	76	29	31	28
3. Infections	135	56	59	54
3.1. Viral infections	47	20	17	16
3.2. Bacterial infections	34	14	11	10
3.3. Fungal infections	8	3	3	3
3.4. Parasite infections	3	1	1	1
4. Malignant neoplasm	23	10	3	3
5. Hypogammaglobulinemia	127	53	59	54
6. (Auto-)inflammation	77	32	35	32
7. CNS manifestation	16	7	9	8
8. Cardiovascular system abnormality	17	7	10	9
Total no. of patients	*n* = 241	100	*n* = 109	100

Since *NFKB1* was reported the most frequent monogenic cause for CVID (6) and pathogenic *NFKB1* variants account for up to 20 % of cases in CVID (12), we analyzed the prevalence of CVID across the entire *NFKB1* cohort. The result of 44% was very similar in both subcohorts, known protein defect and protein defect unclear (analysis not shown).

### Comparison of *NFKB1* variant classes within the known protein defect cohort

As the phenotypic variability of the total *NFKB1* cohort may be caused by the summation of different but distinct phenotypes caused by different types of protein defects in *NFKB1*, we subdivided into more restricted variant classes according to the specific “type” of the underlying protein defect. Due to the limitation of patient numbers in three variant classes, we analyzed five variant classes separately, including carriers of (1) early and (2) central protein truncating variants, (4) early in-frame deletions, (6) protein-decaying missense variants affecting the C-terminal RHD, and (8) large gene deletions.

**Figure 4.**
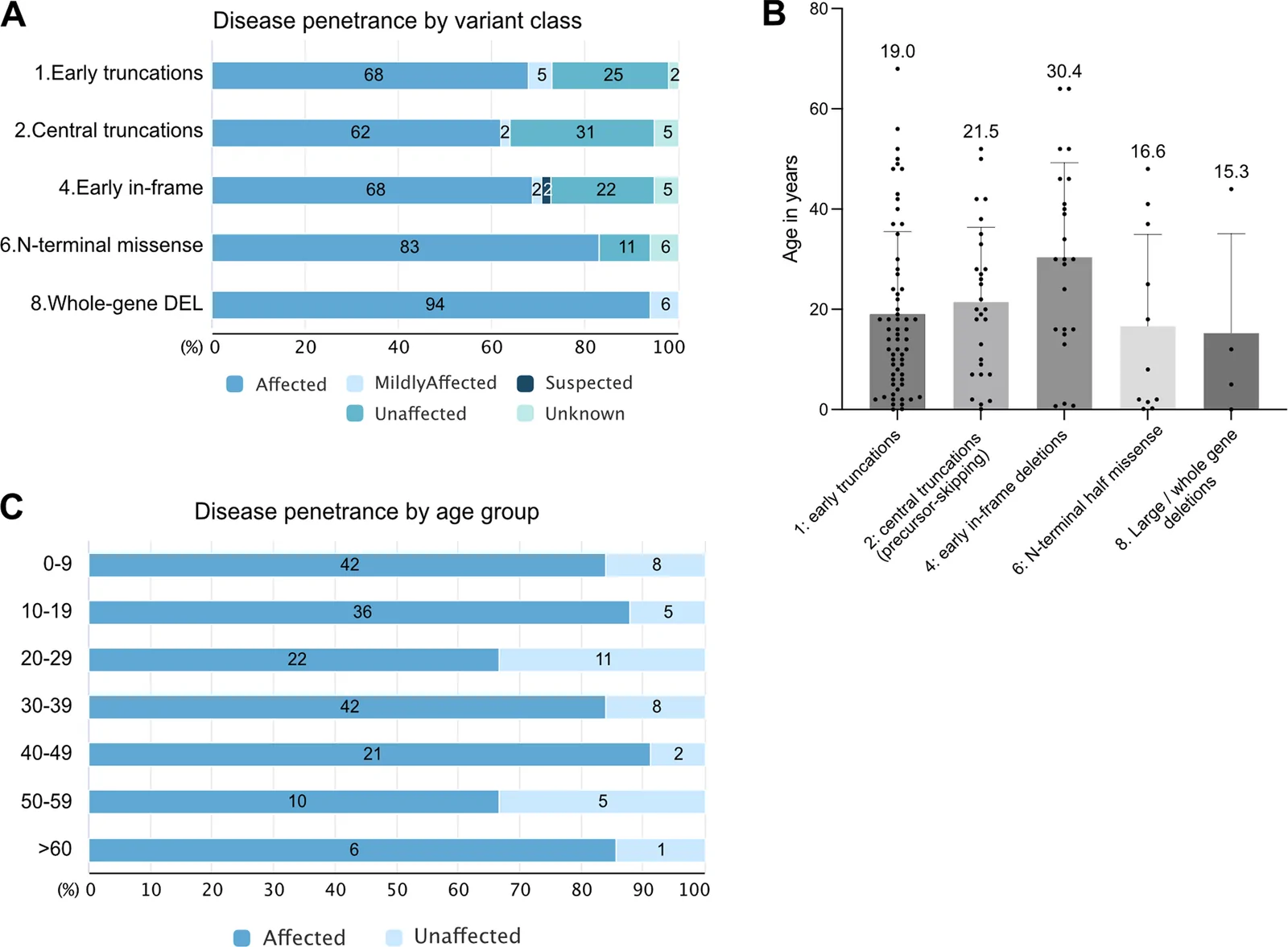
**Age and penetrance characteristics. (A)** Disease penetrance of variant carriers per variant class from the known protein defect cohort, *n*= 241. **(B)** Comparison of AFM between different variant classes, all from the known protein defect cohort, in 241 individuals. **(C)** Age-dependent penetrance in the known protein defect cohort of 241 individuals. Numbers in bars indicate the number of patients per age group; dark bars represent affected individuals; brighter bars represent unaffected but confirmed heterozygous individuals in each age group.

We first calculated the proportion of affected (affected and mildly affected individuals) versus unaffected individuals, which varied significantly between the variant classes, ranging from 67% (central truncations), to 74% (early truncations) and 77% (early in-frame deletions), to 88% (RHD missense variants), up to 100% (complete penetrance) in the large genomic/whole allele deletions (Fig. 4 A). Using multistage Fisher’s exact test, we found that the variant classes differed significantly with regard to their clinical penetrance. Compared with the other variant classes, structural chromosomal aberrations cause pronounced phenotypic abnormalities with complete penetrance. Yet the AFM generally showed a broad range and was not significantly different between the variant classes (Fig. 4 B).

Furthermore, we evaluated whether there is an age-dependent penetrance within the subcohort of variant carriers with known protein defects. The disease penetrance showed itself to be relatively stable throughout all age groups (Fig. 4 C). The oldest patient in our cohort experienced the onset of symptoms at the age of 68 years.

#### Our dataset did not reveal statistically robust phenotype differences between the variant classes

A descriptive analysis of the clinical features across the different variant classes (Table 3) revealed that infections were the most common with 13 cases in 18 individuals with class “6: N-terminal half missense” variants, followed by hypogammaglobulinemia in 12 cases, and lymphoproliferation in 9 of 18 cases. Interestingly, malignant neoplasms occurred rarely, with a maximum of 11 reported patients in class “1: early truncation.” The central truncating variants and early in-frame deletions showed a similar pattern, but with lower absolute numbers, due to the smaller sample size. N-terminal half missense variants showed the highest prevalence of viral infections (33.3 %). Notably, central nervous system (CNS) manifestations and cardiovascular system abnormalities were rare in all genetic variants, suggesting a potentially lower involvement of these organ systems in *NFKB1* insufficiency. Overall, a relatively even distribution of clinical features can be observed, whereas large genomic deletions and whole allele deletion were generally associated with a lower percentage of cases for all clinical features (Table 3). These results can further be confirmed as statistically insignificant in multiple-comparisons Fisher’s exact testing between the underlying five variant classes. Descriptive observations showed that carriers of large deletions more likely had syndromic clinical features, such as facial dysmorphia.

**Table 3.** Crosstable for disease features in variant classes within the known protein defect cohort

Disease manifestation	Early truncations		Central truncations		Early in-frame deletions		N-terminal half missense		Large genomic deletions	
	No. of patients	%	No. of patients	%	No. of patients	%	No. of patients	%	No. of patients	%
1. Autoimmunity	46	41.8	22	40	13	31.7	5	27.8	5	29.4
2. Lymphoproliferation	39	35.5	16	29.1	8	19.5	9	50	4	23.5
3. Infections	67	60.9	27	49.1	23	56.1	13	72.2	5	29.4
3.1. Viral infections	21	19.1	13	23.6	5	12.2	6	33.3	2	11.8
3.2. Bacterial infections	11	10	10	18.2	8	19.5	3	16.7	2	11.8
3.3. Fungal infections	3	2.7	3	5.5	0	0	2	11.1	0	0
3.4. Parasite infections	2	1.8	0	0	1	2.4	0	0	0	0
4. Malignant neoplasm	11	10	3	5.5	5	12.2	2	11.1	2	11.8
5. Hypogammaglobulinemia	58	52.7	25	45.5	27	65.9	12	66.7	5	29.4
6. (Auto-)inflammation	39	35.5	17	30.9	15	36.6	4	22.2	2	11.8
7. CNS manifestation	6	5.5	2	3.6	4	9.8	2	11.1	2	11.8
8. Cardiovascular system abnormality	6	5.5	3	5.5	6	14.6	2	11.1	0	0
Total no. of patients	*n* = 110	​	*n* = 55	​	*n* = 41	​	*n* = 18	​	*n* = 17	​

### Evaluation of exemplary phenotypes for impaired immune regulation—rosacea is so far only reported in patients within the known protein defect cohort

Eight of the 241 individuals in the known protein defect cohort, as well as two of the six cases with unique protein defects from variant class 3, presented with pyoderma gangrenosum. Additionally, one case of pyoderma was identified in cases with unique protein defects from variant class 2. In total, 11 cases (3.3 %) of pyoderma gangrenosum were observed in the entire *NFKB1* insufficiency cohort.

Hepatopathies, including gallbladder pathologies, have been reported in 66 patients, of which we assigned 48 cases to the known protein defect cohort. Most of these cases occurred in variant classes 1 (24 of 110 individuals, 21.8%) or 2 (10 of 55 individuals, 18.2%) or 4 (8 of 41 individuals, 19.5%). Three additional cases each occurred in variant classes 6 (3 of 18, 16.7%) or 8 (3 of 17, 17.7%). The remaining 18 cases were assigned to the protein defect unclear cohort (18 of 109 individuals, 16.5%).

Rheumatological diagnoses such as rheumatoid arthritis, spondyloarthritis, vasculitis, or lupus erythematosus have been reported in *NFKB1*-insufficient patients (*n* = 15). Approximately half (8 cases) of the known cases occurred in patients of the known protein defect cohort, the underlying type of variant appeared to be random. Other cases were observed in the protein defect unclear cohort, and out of the cases with unique protein defects, there was one patient from variant class “2: central truncations” and one from variant class 6: N-terminal half missense variants, respectively, with a rheumatological diagnosis. In contrast, rosacea was reported exclusively in six patients with variants of the known protein defect cohort caused by variants of class 1: early truncations.

### Separate descriptive evaluation of heterozygous variant carriers within large deletions

This study includes 17 patients reported with heterozygous large genomic deletions involving *NFKB1,* who were therefore all assumed to have lost expression from the affected allele (Table S1). Since all 17 individuals were affected (including one mildly affected individual), penetrance is complete in this variant class. For four patients, AFM was reported with 0, 5, 12, and 44 years, respectively, and one patient deceased at the age of 52 years. Among the available 14 clinical case reports, seven individuals were described to have syndromic clinical features, such as global developmental delay, facial dysmorphism, and intellectual disability, while three individuals had a diagnosis of CVID or a CVID-like disease (*n* = 3 of 17; 18 %). Autoimmunity, abnormal lymphoproliferation, hypogammaglobulinemia, and/or recurrent infections were reported in seven cases, consistent with the most frequent disease manifestations typically associated with pathogenic *NFKB1* variants.

## Discussion

Here, we provide a reference work compiling all hitherto reported individuals carrying *NFKB1* variants, their clinical course, and their family histories. The summarized and curated information is accessible in the Supplementary Materials (Table S1) and is freely available and searchable online via the GenIA database website (https://www.geniadb.net).

*NFKB1* insufficiency is associated with various clinical features, and its phenotype varies in each individual patient. Progressive disease courses (characterized by a worsening of symptoms over time and the emergence of additional clinical manifestations) were observed for most of the affected individuals. The mean age of onset in the total cohort was 19 years under the premise of a wide variability. The AFM ranged from birth to 68 years. However, a large proportion (74 of 350; 21%) of carriers of the total cohort have been reported as asymptomatic and additional 13 carriers were reported with only mild symptoms.

Although the clinical manifestations in patients with *NFKB1* variants appear highly heterogeneous at first glance, the overall constellation of the most frequent clinterms across different variant classes reveals a remarkable degree of similarity and uniformity, with only the order of their frequency varied. Most patients exhibited a combination of hypogammaglobulinemia, recurrent (respiratory tract) infections, and autoimmunity (such as autoimmune cytopenias). This clinical pattern was observed in both subcohorts. Individuals in the known protein defect cohort, however, had a statistically higher frequency of both malignancies and autoimmune disorders. As no clear patterns emerge in the specific autoimmune or malignant disease entities, and the exact case numbers do not differ substantially, the interpretation as type I error appears more plausible than a systematic difference. Compared with the other variant classes, structural chromosomal aberrations caused more likely syndromic clinical features, such as facial dysmorphia, and had a complete disease penetrance.

One of the predominant manifestations of heterozygous pathogenic *NFKB1* variants is hypogammaglobulinemia, mainly presenting as CVID, and, vice versa, pathogenic *NFKB1* variants represent the most frequent monogenic cause of CVID (6, 12). It was diagnosed in 107 individuals (equals 44 %) of all 241 variant carriers in the known protein defect cohort.

In this study, we analyzed whether there is a genotype–phenotype correlation in *NFKB1*-mutated individuals from a worldwide, retrospective literature acquisition. Since the first affected families had been reported, it became evident that heterozygous variants in *NFKB1* may produce a highly heterogeneous disease phenotype with incomplete penetrance, even within one family carrying the very same pathogenic variant in *NFKB1.* Given this considerable biological heterogeneity, it is remarkable that relatively comparable clinical phenotypes emerge across different genotypes. Among the *NFKB1*-variant carriers, we asked whether the clinical features of *NFKB1* insufficiency correlate with the underlying variant and hence the biochemical protein defect, similar as recently described for *NFKB2* (13).

Even though the clinical manifestations in patients with *NFKB1* variants are highly heterogeneous, the 10 most frequent overarching clinterms were remarkably similar in all variant cohort subgroups, with only minor differences in their frequency. The diversity of the phenotypic presentations is based on the individual combination of hypogammaglobulinemia, recurrent (respiratory tract) infections, and autoimmunity (such as autoimmune cytopenias). This remarkably constant combinatorial clinical pattern appears to occur independent of the underlying variant type, suggesting a shared pathophysiology that transcends the molecular heterogeneity.

We hypothesized that the phenotypic heterogeneity is at least partially explained by an underlying genotypic diversity. However, we did not observe any meaningful difference between the phenotypes of our different variant classes, indicating that additional (unknown) variants and/or environmental and/or epigenetic factors may influence the variable expressivity and the reduced penetrance in patients with variants in *NFKB1*.

The frequently observed progressive disease course in individuals carrying pathogenic *NFKB1* variants is generally characterized by a worsening of symptoms over time and the emergence of additional clinical manifestations, such as autoinflammation or autoimmunity. Yet the autoinflammatory component of the disease can be controlled with, e.g., anti-TNF-a or anti-IL-1 therapeutic monoclonal antibodies, which dampens overactive canonical NF-κB (14, 15). In patients with a severe and recalcitrant clinical course, hematopoietic stem cell transplantation may be considered. Comprehensive treatment data remain limited; hence, a further retrospective cohort study, initiated by the last author, will address management outcomes in *NFKB1*-insufficient patients.

Our results may be useful for physicians taking care of patients with a known pathogenic *NFKB1* variant, and in particular of newly diagnosed patients, as well as for families seeking genetic counseling. Yet, because of the highly heterogeneous disease character, predictions regarding disease progression and outcome should be considered with caution. For instance, although the statistical mean age of onset in the total *NFKB1* cohort is 19 years, its range is from birth to 68 years.

To date, Mandola et al. (16) have published the only case of a homozygous variant in *NFKB1*. In this family, the parents and two obligate carriers of the variant in heterozygosity were described as completely healthy. The observed immune deviations—including IκB-α impairment as seen in the published patient—are not unique to *NFKB1* variants and can actually be seen in other patients with variants upstream of *NFKB1* or patients with an unknown/polygenic cause of immunodeficiency.

A limitation of our study is the retrospective nature of the data collection and the sometimes incomplete dataset, e.g., when only major clinical symptoms were reported or laboratory values were not accessible. Therefore, we contacted the original authors of the reports whenever possible to gather additional information.

Longitudinal data are usually not available, since patients and their families have only occasionally been reported in follow-up studies. Two initially healthy family members were confirmed as heterozygous carriers and became affected later in life, explaining their negative value for the diagnostic delay. In addition, it remains conceivable that the reported penetrance is influenced by the study design (i.e., *NFKB1* families with no affected family member would not be reported or registered).

Furthermore, our analysis highlights the importance of careful data validation, as almost half of the published *NFKB1* variant carriers have been described in more than one independent study, without being clearly indicated in some cases, emphasizing the need for meticulous cohort assessment in future studies. Utilization of unique patient identifiers would generally prevent errors, such as duplicate data entry. As far as possible, we harmonized redundant and/or updated information for each variant carrier upon data collection in the GenIA and made the datasets easily available as a reference database and as a source for future statistical studies.

Some variant classes appeared to be particularly rare, with the corresponding number of individuals being too small to allow for statistical assessments of differences, which therefore may have escaped our current analysis. Hence, further investigation with an even larger cohort size in a long-term and prospective study for consideration of potential confounding factors is warranted to corroborate our findings and explore subtle differences that could have important clinical implications.

In the context of the variety of the inferred protein defects, it therefore remains surprising that no clear genotype–phenotype association is distinguishable. It seems likely that disease onset, progression, and clinical phenotypes are dependent on epigenetic or environmental disease modifiers or secondary pathomechanisms that are yet to be identified. Different pathomechanisms could occur depending on whether signaling molecules are simply insufficient (loss of expression) or whether mutant proteins (gain/loss-of-function) are subject to secondary decay, e.g., due to misfolding. Furthermore, a recent publication described the potential inflammatory effects occurring by a quantitative p50 deficiency through a reduced feedback loop from over all lower levels of p50 homodimers (17). However, for the time being, the causalities of both the reduced penetrance and the variable expressivity remain enigmatic.

## Materials and methods

### Literature review and data storage using the publicly accessible GenIA database

We performed a retrospective review on *NFKB1*-associated publications up to December 2024 containing genotypic and phenotypic information on patients or families reported to carry *NFKB1* variants or papers containing experimental and functional data on *NFKB1* variants (Fig. 2 A and Table S4). Furthermore, this study presents previously undescribed variants identified in patients followed at the CCI, University of Freiburg, Germany. To be compliant with relevant data protection regulations and ethical guidelines, all data used in this study were collected either from published reports or from clinical records following double-pseudonymization. Data were collected and harmonized using the publicly accessible GenIA database (https://www.geniadb.net), where all information is accessible. GenIA is an international collaborative effort that aims to catalog all relevant immunogenetics knowledge. Its database is designed for the systematic collection and comprehensive evaluation of clinical, laboratory, genetic, and functional data from patients with genetically driven immune disorders. The narrative literature search was performed in PubMed using different keyword combinations including *NFKB1*, *NF-κB*,* immunodeficiency*, *inflammation*, *autoimmunity*, *patient*, *case report*, *variant*, *CVID*, *mutation*, and/or *phenotype*. This literature review, conducted up to December 2024, identified 70 relevant publications (Fig. 2 A and Table S4) (3, 4, 6, 8, 9, 10, 11, 12, 14, 15, 17, 18, 19, 20, 21, 22, 23, 24, 25, 26, 27, 28, *Preprint*, 16, 29, 30, 31, 32, 33, 34, 35, 36, 37, 38, 39, 40, 41, 42, 43, 44, 45, 46, 47, 48, 49, 50, 51, 52, 53, 54, 55, 56, 57, 58, 59, 60, 61, 62, 63, 64, 65, 66, 67, 68, 69, 70, 71, 72, 73, 74, 75), which included original research articles, letters, case reports, review articles, and one journal abstract. Two publications were excluded because the two reported individuals carried somatic *NFKB1* variants in cancer cells (42, 71) (c.419T>A; p.Leu140Gln; L140Q and c.1289G>A; p.Gly430Glu; G430E). Another publication reporting the first case of a homozygous germline *NFKB1* variant within a consanguineous family was also excluded because of uncertain evidence of pathogenicity (16) (c.2878G>A; p.Gly960Arg; G960R).

### Assembling the cohort

After reviewing the 67 relevant papers, we documented information on 815 individuals into GenIA (Table S1). Of these 815 individuals, 333 were proven carriers of heterozygous *NFKB1* variants, 17 were obligate carriers, and 19 were presumed carriers of these variants, and 446 were either *NFKB1* wild-type relatives or untested relatives. This cohort of 815 individuals included 767 previously reported and 48 so far unreported individuals, including patients and their relatives seen either at the CCI at the Medical Center University of Freiburg, or through contacts with authors (Table S2). When individuals were reported twice or more, we linked them across the respective papers in GenIA (Fig. 2 B). We collected the following information for all individuals: sex, AFM, age at genetic diagnosis, ethnicity, parental concordance, the *NFKB1* gene variant, Human Phenotype Ontology–compatible phenotypic/clinical features, treatment outcomes, and specific laboratory values, such as cell counts and immunoglobulin levels. In cases, when lab values were reported without their respective reference range, we used reference ranges from published research studies on healthy populations, the results of which were also stored in the GenIA database (https://geniadb.org). In addition, we collected the available information on all family members including sex, family tree position/degree of relationship, ethnicity, disease status, consanguinity, current age, age at genetic diagnosis, AFM, and, if applicable, age at death. Where applicable, additional information was requested from the authors and kindly provided.

Pedigree analyses allowed us to identify obligate or presumed variant carriers who were not genetically tested. Obligate carriers are relatives of patients with known NF-κB1 insufficiency in whom, based on an evaluation of the pedigree, variant carriage can be assumed with near-certainty. The presence or absence of clinical symptoms was irrelevant for the classification as an obligate carrier. Presumed carrier relatives carry a known variant in *NFKB1* and are also known to have experienced symptoms during their lifetime consistent with NF-κB1 insufficiency albeit they were not genetically evaluated (e.g., deceased individual). Gonadal mosaicism was either not tested or could therefore not be confirmed.

### Categorization of clinterms and grouping of disease features

The collected data were not always reported with the same level of accuracy, and in several cases, we contacted the authors to request additional or more detailed phenotypic information. We introduced the neologism clinterm as an umbrella term that collectively refers to clinical phenotypes, features, signs, manifestations, symptoms, and diagnoses to streamline and simplify clinical discourse. Furthermore, a patient may have multiple symptoms simultaneously and therefore fall into multiple categories.

However, we encountered more than 600 different clinterms and their subgroups with limited comparability. To address this issue, an initial summarization of “clinterm groups” was performed with the aim of identifying the 10 most frequently mentioned clinterms. For this purpose, existing clinterms were used as overarching supercategories, to which all related, more specific clinterms were assigned (Text S1). The suffix “gr” was used to denote these groupings.

To develop a correlation of the phenotypes with the underlying p105/p50 protein defects, we generated in a second step groups of disease features, to enable analyses aligned with a practical medical perspective. We defined eight new clinically justified categories, with generalized designations, allowing for a stronger focus on clinically relevant disease features: (1) autoimmunity, (2) lymphoproliferation, (3) infection, (4) malignant neoplasm, (5) hypogammaglobulinemia, (6) (auto-)inflammation, (7) CNS manifestations, and (8) cardiovascular system abnormality (Text S2). In addition, we subdivided the group of infections into bacterial, viral, fungal, and protozoan infections. As a correlation of pyoderma gangrenosum and underlying *NFKB1* variants had been suggested (36, 65), the presence of pyoderma gangrenosum in the cohort was examined descriptively. In addition, based on our clinical observations at the University of Freiburg, the following phenotypes were analyzed descriptively: rosacea, hepatopathies, and rheumatological diagnoses.

### Variant classification and allocation into “protein defect” subcohorts

In total, we identified in the cohort of 333 *NFKB1* variant carriers 157 distinct *NFKB1* variants, which predict a broad variety of resulting protein defects (if the deduced mutant proteins are expressed at all). Thus, we hypothesized that the phenotypic heterogeneity partially reflects the underlying diversity of genetic defects.

Initially, we examined potential comparison between the variants confirmed or assumed to be pathogenic or likely pathogenic from those that do not or whose effect of the resulting protein(s) cannot be tested. The currently available experimental in vitro tests (3, 4, 5, 6) do not cover all p105 and p50 functions. In general, only the damaging effects can be affirmed with some accuracy, whereas benignity cannot be verified. While the outcome of short truncating variants appears to be solved, the impact of central and late truncating variants, if they all result in protein expression, is unclear. Missense variants need to be tested individually, but the role of isomorphic p50 variants and of variants predominantly affecting p105-specific functions still remains obscure. In total, the known protein defect cohort includes nonsense variants, missense variants with functional evidence from in vitro tests, and splice site variants with evidence for alternative splicing. The remaining variants with unknown, unpredictable, and isomorphic effects, and cases with unique protein defects were summarized in the “*NFKB1* variant with unknown protein defect” cohort (Tables S1 and S3).

In a next step, the cohort was divided into the above-mentioned eight variant classes, and a multigroup comparison was subsequently performed, including the “variants with known protein defects” and large genomic deletions. According to the hypothetic protein defect deduced from the corresponding genomic variant, we assigned the variant carriers into the following eight categories (Fig. 1 and Tables S1 and S3) representing the eight variant classes: (1) “early” truncations, which result from nonsense or frameshift variants, typically on the N-terminal side relative to the nuclear localization sequence, and cause loss of p105 and p50 expression; (2) “central” truncations, which predict the immediate expression of p50-like proteins instead of being generated from their p105 precursors (“precursor skipping”); (3) “late” truncations, which affect only the C-terminal domains of p105; (4) “early” in-frame deletions, mainly resulting from splicing errors, which can cause expression of mutant precursors which, however, undergo accelerated decay instead of being processed to p50; (5) “late” in-frame deletions, which are particularly rare but could be converted to p50, if processing is enabled; (6) missense variants residing within the N-terminal “p50 half,” including the frequent protein-decaying variants and sporadic hypomorphic and loss-of-function variants with normal protein stability; (7) missense variants residing within the C-terminal half of p105 and most likely affecting only p105-specific functions; and (8) large genomic deletions involving the entire *NFKB1* or parts thereof.

### Statistical analyses

For data analysis, we first assessed the suitability of parametric assumptions by evaluating normality (Shapiro–Wilk test) and applied the Kruskal–Wallis test, Mann–Whitney U test, and Fisher’s exact test. Post hoc tests were conducted with Fisher’s exact test for multiple column comparison. Because all analyses were exploratory and P values and 95% confidence intervals were not corrected for multiple comparisons, inferences may not be reproducible. Statistical analysis of data was performed considering the distribution characteristics and the study design to ensure valid and reproducible results. Statistical calculations were performed in Excel and GraphPad Prism, and figures were prepared using BioRender and Affinity.

### Online supplemental material

Fig. S1 shows the 10 most often reported simple clinterms for the known protein defect subcohort, *n* = 241; the 10 most often reported simple clinterms for the protein defect unclear subcohort, *n* = 109; and CVID diagnosis for each variant class. Table S1 shows all 815 individuals of the study. Table S2 shows new cases (blue) with their respective family members (black) in this study. Table S3 shows the 157 distinct variants of *NFKB1* were included in this study, categorized according to the subcohorts and structured by their deduced protein defect. Table S4 shows the list of inspected and included papers. Table S5 shows the list of variants of uncertain significance (VUS) from gnomAD with variants in this study (*n* = 157). Table S6 shows the list of VUS from gnomAD with allele frequency <0.00001. Table S7 shows the list of VUS from gnomAD with allele frequency 0.01–0.00001. Table S8 shows the list of VUS from gnomAD with allele frequency >0.01. Data S1 shows generated pedigrees, stating the original article PMID and publication year. Text S1 shows the list of supercategories of clinterms. Text S2 shows the disease features list.

## Supplementary Material

Table S1 shows all 815 individuals of the study.

Table S2 shows new cases (blue) with their respective family members (black) in this study.

Table S3 shows the 157 distinct variants of *NFKB1* were included in this study, categorized according to the subcohorts and structured by their deduced protein defect.

Table S4 shows the list of inspected and included papers.

Table S5 shows the list of VUS from gnomAD with variants in this study (*n* = 157).

Table S6 shows the list of VUS from gnomAD with allele frequency <0.00001.

Table S7 shows the list of VUS from gnomAD with allele frequency 0.01–0.00001.

Table S8 shows the list of VUS from gnomAD with allele frequency >0.01.

Data S1 show familial cases with generated pedigrees, stating the original article PMID and publication year.

Text S1 shows the list of supercategories of clinterms.

Text S2 shows the disease features list.

## Data Availability

The data underlying Figs. 1, 2, 3, and 4 are available in the published article and its online supplemental material.
