# Supplementary material for: Genotype–phenotype analysis in 333 individuals with heterozygous variants in NFKB1
Source: J Hum Immun. 2026 Oct 19;3(1):e20260076. doi: 10.70962/jhi.20260076 (PMC13679715; doi:10.70962/jhi.20260076)
Supplement: Supplementary file 1 [file jhi_20260076_datas1.docx]

**Data S1.** Familial cases with generated pedigrees, stating the original article PMID and publication year
