# Supplementary material for: Genotype–phenotype analysis in 333 individuals with heterozygous variants in NFKB1
Source: J Hum Immun. 2026 Oct 19;3(1):e20260076. doi: 10.70962/jhi.20260076 (PMC13679715; doi:10.70962/jhi.20260076)
Supplement: Supplementary file 2 [file jhi_20260076_datas2.docx]

**Text S1.** Summarization of disease groups as overarching categories (supercategories)

gr = stands for group

Autoimmunity gr

Atopic dermatitis

Arteritis

Autoimmune antibody positivity

Autoimmune cytopenia

Autoimmune enteropathy

Autoimmune hemolytic anemia

Autoimmune myocarditis

Autoimmune neutropenia

Autoimmune thrombocytopenia

Autoimmune thyroiditis

Autoimmunity

Celiac disease

Coombs-positive hemolytic anemia

Diabetes mellitus

Morphea

Multiple Sclerosis

Psoriasiform dermatitis

Pyoderma

Pyoderma gangrenosum

Type I diabetes mellitus

Vitiligo

anti-TPO antibodies

Anticardiolipin IgG antibody positivity

Antineutrophil antibodies

Antinuclear antibodies

Antiphospholipid antibody positive

Coombs Test Positive

Coombs-positive hemolytic anemia

Platelet antibody positive

Rheumatoid factor positive

Systemic Autoinflammation gr

Aphthous oral ulcer

Behcet disease

Hepatitis

Keratitis

Lymphoid interstitial pneumonia

Mucositis

Necrotizing cellulitis

Oral ulcer

Orbital cellulitis

Parotitis

Pericarditis

Polymyalgia Rheumatica

Psoriasiform dermatitis

Psoriatic Arthritis

Pyoderma

Pyoderma gangrenosum

Rheumatoid arthritis

Sjogren Syndrome

Small vessel vasculitis

Spondyloarthritis

Stomatitis

Systemic lupus erythematosus

Vasculitis

Psoriatic Arthritis gr

Psoriasiform dermatitis

Autoimmune cytopenia gr

Autoimmune hemolytic anemia

Autoimmune neutropenia

Autoimmune thrombocytopenia

Coombs Test Positive

Coombs-positive hemolytic anemia

Coombs-positive hemolytic anemia gr

Anemia

Autoimmune hemolytic anemia

Coombs Test Positive

Hemolytic anemia

Autoimmune thrombocytopenia gr

Thrombocytopenia

Autoimmune neutropenia gr

Neutropenia

Anemia gr

Aplastic anaemia

Autoimmune hemolytic anemia

Coombs-positive hemolytic anemia

Hemolytic anemia

Hypochromic microcytic anemia

Iron deficiency anemia

Microcytic anemia

Pancytopenia

Pernicious anemia

Autoimmune antibody positivity gr

anti-TPO antibodies

Anticardiolipin IgG antibody positivity

Antineutrophil antibodies

Antinuclear antibodies

Antiphospholipid antibody positive

Coombs Test Positive

Coombs-positive hemolytic anemia

Platelet antibody positive

Rheumatoid factor positive

Arthritis gr

Arthropathy

Oligoarthritis

Polyarthritis

Psoriatic Arthritis

Reactive arthritis

Rheumatoid arthritis

Spondyloarthritis

Osteoarthritis gr

Hip osteoarthritis

Knee osteoarthritis

Nervous system abnormality gr

Bacterial meningitis

Behavioral abnormality

Brachial plexus neuropathy

cerebral granulomatosis

Cerebral infarct

Cerebral vasculitis

Chronic CSF lymphocytosis

Delirium

Depressive disorder

Facial palsy

Headaches

Ischemic stroke

Meningitis

Migraine

Migraine with aura

Multiple Sclerosis

Myelitis

Non-infectious meningitis

Optic neuritis

optic papillitis

Paresthesia

Progressive leukoencephalopathy

Progressive multifocal leukoencephalopathy

Restless legs syndrome

Retinitis

Seizures

Subdural hemorrhage

Tremor

Viral meningitis

Migraine gr

Migraine with aura

optic papillitis gr

Optic neuritis

Meningitis gr

Bacterial meningitis

Non-infectious meningitis

Viral meningitis

Recurrent infections gr

Cytomegalovirus Infection Reactivation

Herpes labialis, recurrent

Recurrent aphthous stomatitis

Recurrent bronchitis

Recurrent ear infections

Recurrent fungal infections

Recurrent Haemophilus influenzae infections

Recurrent lower respiratory tract infections

Recurrent mycobacterium avium complex infections

Recurrent opportunistic infections

Recurrent pharyngitis

Recurrent shingles

Recurrent streptococcus pneumoniae infections

Recurrent upper respiratory tract infection

Recurrent viral infections

repeated bacterial infections

Unusual infection gr

(unusual) Epstein-Barr virus infection

(unusual) Fungal infection

(unusual) Giardiasis

(unusual) Viral infection

Chronic furunculosis

Cutaneous abscess

Cutaneous fungal infections

Cytomegalovirus colitis

Cytomegalovirus infection

Cytomegalovirus Infection Reactivation

Disseminated nontuberculous mycobacterial infection

Invasive pulmonary aspergillosis

Liver abscess

Lung abscess

Mucocutaneous candidiasis

Pleural empyema due to Salmonella

Pneumocystis jiroveci pneumonia

Progressive leukoencephalopathy

Protozoan infection

Recurrent aphthous stomatitis

Recurrent bronchitis

Recurrent Haemophilus influenzae infections

Recurrent infections

Recurrent lower respiratory tract infections

Recurrent mycobacterium avium complex infections

Recurrent opportunistic infections

Recurrent shingles

Recurrent streptococcus pneumoniae infections

Recurrent upper respiratory tract infection

Recurrent viral infections

repeated bacterial infections

Severe SARS-CoV-2 infection

Unusual gastrointestinal infection

(unusual) Viral infection gr

(unusual) Epstein-Barr virus infection

(unusual) influenza infection

Cytomegalovirus colitis

Cytomegalovirus infection

Cytomegalovirus Infection Reactivation

Genital herpes

Hepatitis B

Hepatitis C

Herpes labialis, recurrent

Herpes Simplex Virus Infection

HIV Infection

Human papilloma virus infection

Measles

Molluscum contagiosum

Mumps

Neisseria meningitidis infection

Norovirus infection

Parvovirus infection

Persistent CMV viremia

Plantar warts

Progressive leukoencephalopathy

Progressive multifocal leukoencephalopathy

Recurrent shingles

Recurrent viral infections

Rotavirus infection

RSV pneumonia

Rubella

Severe SARS-CoV-2 infection

Stomatitis

Subglottic laryngitis

Varicella zoster virus infection

Viral hepatitis

Viral meningitis

Warts

Stomatitis gr

Recurrent aphthous stomatitis

(Unusual) bacterial infection gr

Bacterial mastitis

Bacterial meningitis

Campylobacter infection

Cellulitis

Chronic furunculosis

Clostridium difficile colitis

Cutaneous abscess

Disseminated nontuberculous mycobacterial infection

Epiglottitis

Erysipelas

Folliculitis

Furuncle

Gluteal abscess

Helicobacter pylori infection

Liver abscess

Lung abscess

Mycobacterium Tuberculosis infection

Necrotizing Tonsillitis

Neonatal omphalitis

Parapharyngeal abscess

Paronychia

Perianal Abscess

Periodontitis

Pleural empyema

Pleural empyema due to Salmonella

Recurrent Haemophilus influenzae infections

Recurrent mycobacterium avium complex infections

Recurrent streptococcus pneumoniae infections

repeated bacterial infections

Salmonella infection

Streptococcal tonsillitis

Suppurative Otitis Media

Syphilis

Tooth abscess

Urinary tract infection

(unusual) Fungal infection gr

Cutaneous fungal infections

Esophageal candidiasis

Invasive pulmonary aspergillosis

Mucocutaneous candidiasis

Onychomycosis

Oral candidiasis

Pneumocystis jiroveci pneumonia

Recurrent fungal infections

Vulvovaginal candidiasis

Protozoan infection gr

(unusual) Giardiasis

Lung disease gr

(unusual) influenza infection

Allergic asthma

Asthma

Bronchiectasis

Bronchitis

Chronic lung disease

Chronic obstructive pulmonary disease

Cough

Granulomatous-lymphocytic interstitial lung disease

Interstitial lung disease

Invasive pulmonary aspergillosis

Lung abscess

Lung adenocarcinoma

Lymphoid interstitial pneumonia

obstructive lung disease

Pleural effusion

Pleural empyema

Pleural empyema due to Salmonella

Pneumocystis jiroveci pneumonia

Pneumonia

Pneumothorax

Pulmonary embolism

Pulmonary Emphysema

Pulmonary fibrosis

Pulmonary granulomatosis

Pulmonary Hypertension

Pulmonary insufficiency

Pulmonary nodule

Recurrent bronchitis

Recurrent lower respiratory tract infections

Recurrent upper respiratory tract infection

Respiratory failure

Respiratory insufficiency

Restrictive ventilatory defect

RSV pneumonia

Severe SARS-CoV-2 infection

Sinopulmonary infections

Asthma gr

Allergic asthma

Chronic lung disease gr

Chronic obstructive pulmonary disease

obstructive lung disease gr

Chronic obstructive pulmonary disease

Asthma

Allergic asthma

(unusual) Respiratory tract infection gr

(unusual) influenza infection

Bronchitis

Chronic rhinitis

Epiglottitis

Invasive pulmonary aspergillosis

Lung abscess

Parapharyngeal abscess

Pharyngalgia

Pharyngitis

Pleural empyema

Pleural empyema due to Salmonella

Pneumocystis jiroveci pneumonia

Pneumonia

Purulent rhinitis

Recurrent bronchitis

Recurrent lower respiratory tract infections

Recurrent pharyngitis

Recurrent upper respiratory tract infection

Rhinitis

RSV pneumonia

Severe SARS-CoV-2 infection

Sinopulmonary infections

Sinusitis

Subglottic laryngitis

Tonsillitis

Pharyngitis gr

Recurrent pharyngitis

Rhinitis gr

Chronic rhinitis

Purulent rhinitis

Pneumonia gr

Lymphoid interstitial pneumonia

Pneumocystis jiroveci pneumonia

RSV pneumonia

Hepatitis gr

Hepatitis

Elevated hepatic transaminases

Hepatic granulomatosis

Hepatitis B

Hepatitis C

Viral hepatitis

Viral hepatitis gr

Hepatitis B

Hepatitis C

Hepatopathy gr

Biliary cholangitis

cirrhosis

Elevated hepatic transaminases

Hepatic failure

Hepatic fibrosis

Hepatic granulomatosis

Hepatic steatosis

Hepatitis

Hepatitis B

Hepatitis C

Hepatomegaly

Hepatosplenomegaly

Liver abscess

Nodular regenerative hyperplasia of liver

Portal hypertension

Portal vein thrombosis

Viral hepatitis

Lymphadenopathy gr

Cervical lymphadenopathy

Enlarged mesenteric lymph node

Follicular hyperplasia

Intestinal lymphoid nodular hyperplasia

Lymph node abscess

Lymphadenitis

Lymphoproliferative disorder

Mediastinal granulomatous lymphadenitis

Mediastinal lymphadenopathy

Hepatosplenomegaly gr

Hepatomegaly

Splenomegaly

Abnormal weight loss gr

Decreased body mass index

Abnormal lymphocyte proliferation gr

B-cell lymphoma

Cervical lymphadenopathy

Chronic lymphatic leukemia

Enlarged mesenteric lymph node

Follicular hyperplasia

Follicular lymphoma

Generalized lymphadenopathy

Granulomatous-lymphocytic interstitial lung disease

Hepatomegaly

Hepatosplenomegaly

Intestinal lymphoid nodular hyperplasia

Large granular lymphocytic leukemia

Lymphadenitis

Lymphadenopathy

Lymphocytic colitis

Lymphoid hyperplasia

Lymphoid interstitial pneumonia

Lymphoproliferative disorder

Mediastinal granulomatous lymphadenitis

Mediastinal lymphadenopathy

Nodular regenerative hyperplasia of liver

Non-Hodgkin lymphoma

Splenomegaly

T-cell lymphoma

Lymphadenitis gr

Mediastinal granulomatous lymphadenitis

Skin abnormality gr

Abscess

Acute cutaneous wound

Alopecia

Alopecia areata

Alopecia totalis

Anal fistula

Angioedema

Aphthous oral ulcer

Atopic dermatitis

Basal cell carcinoma

Cafe-au-lait spot

Cellulitis

Chronic furunculosis

Cutaneous abscess

Cutaneous fungal infections

Ecchymosis

Eczema

Erysipelas

Erythema

erythema nodosum

Fistula

Folliculitis

Furuncle

Genital herpes

Genital ulcers

Gluteal abscess

Herpes labialis, recurrent

Herpes Simplex Virus Infection

Human papilloma virus infection

Hyperkeratosis

Hyperpigmentation of the skin

Lichen Planus

Molluscum contagiosum

Morphea

Mucocutaneous candidiasis

Mucositis

Nail dystrophy

Necrotizing cellulitis

Necrotizing fasciitis

Neutrophilic infiltration of the skin

Onychomycosis

Oral leukoplakia

Oral ulcer

Paronychia

Perianal Abscess

Petechiae

Plantar warts

Poor wound healing

Pruritus

Psoriasiform dermatitis

Pyoderma

Pyoderma gangrenosum

Raynaud phenomenon

Rosacea

Skin hypopigmentation

Skin infections

Skin rash

Squamous cell carcinoma

Sterile abscess

Urticaria

Vaginal mucosal ulceration

Vaginitis

Vitiligo

Warts

Genital ulcers gr

Vaginal mucosal ulceration

Pyoderma gangrenosum gr

Pyoderma

Aphthous oral ulcer gr

Oral ulcer

Furuncle gr

Chronic furunculosis

Fistula gr

Anal fistula

Esophagitis gr

Eosinophilic esophagitis

Warts gr

Plantar warts

Skin infections gr

Abscess

Cellulitis

Chronic furunculosis

Cutaneous abscess

Cutaneous fungal infections

Erysipelas

Folliculitis

Furuncle

Genital herpes

Gluteal abscess

Herpes labialis, recurrent

Human papilloma virus infection

Molluscum contagiosum

Mucocutaneous candidiasis

Necrotizing cellulitis

Onychomycosis

Perianal Abscess

Plantar warts

Skin abnormality

Vaginitis

Warts

Cellulitis gr

Necrotizing cellulitis

Orbital cellulitis

Herpes Simplex Virus Infection gr

Herpes labialis, recurrent

Genital herpes

Atopy gr

Atopic dermatitis

Kidney abnormality gr

Acute kidney failure

Chronic kidney disease

Decreased urine output

Lupus nephritis

Nephromegaly

Nephrotic syndrome

proteinuria

Pyelonephritis

Renal angiomyolipoma

Renal neoplasm

Renal transitional cell carcinoma

Stage 3 chronic kidney disease

Alopecia gr

Alopecia areata

Alopecia totalis

Malignant neoplasm gr

B-cell lymphoma

Basal cell carcinoma

Breast carcinoma

cervical carcinoma

Chronic lymphatic leukemia

Follicular lymphoma

Large granular lymphocytic leukemia

Lung adenocarcinoma

Non-Hodgkin lymphoma

Renal neoplasm

Renal transitional cell carcinoma

Squamous cell carcinoma

Squamous cell carcinoma of the tongue

T-cell lymphoma

Abscess gr

Cutaneous abscess

Gluteal abscess

Liver abscess

Lung abscess

Lymph node abscess

Pancreatic abscess

Parapharyngeal abscess

Perianal Abscess

Peritoneal abscess

Sterile abscess

Tooth Abscess

Vasculitis gr

Cerebral vasculitis

Small vessel vasculitis

Arteritis

Abnormality of thyroid physiology gr

anti-TPO antibodies

Autoimmune thyroiditis

Graves disease

Hyperthyroidism

Hypothyroidism

Thyroid gland abnormality gr

Abnormality of thyroid physiology

anti-TPO antibodies

Autoimmune thyroiditis

Graves disease

Hyperthyroidism

Hypothyroidism

Diabetes mellitus gr

Type I diabetes mellitus

Type II diabetes mellitus

Drug-induced diabetes mellitus

Lactose intolerance gr

Decreased small intestinal mucosa lactase level

Abdominal pain gr

Abdominal cramps

Diarrhea gr

Bloody diarrhea

Clostridium difficile colitis

Intermittent diarrhea

Colitis gr

Clostridium difficile colitis

Cytomegalovirus colitis

Lymphocytic colitis

Microscopic colitis

Pancolitis

ulcerative colitis

Abnormal size of nasopharyngeal adenoids gr

Increased size of nasopharyngeal adenoids

Mucocutaneous candidiasis gr

Esophageal candidiasis

Oral candidiasis

Vulvovaginal candidiasis

Septicaemia gr

Neonatal sepsis

Sterile sepsis

Allergy gr

Allergic asthma

Allergic rhinitis

Antibiotic allergy

Asthma

Drug allergy

Nut food product allergy

Penicillin Allergy

Pollen allergy

Drug allergy gr

Antibiotic allergy

Penicillin Allergy

Hypogammaglobulinemia gr

Agammaglobulinemia

Decreased circulating IgG subclass level

Decreased circulating level of specific antibody

Decreased IgA levels

Decreased IgE levels

Decreased IgG levels

Decreased IgM levels

Panhypogammaglobulinemia

Reduced IgG1 levels

Reduced IgG2 levels

Reduced IgG3 levels

Reduced IgG4 levels

Fever gr

Recurrent fever

Episodic fever
