# Supplementary material for: Genotype–phenotype analysis in 333 individuals with heterozygous variants in NFKB1
Source: J Hum Immun. 2026 Oct 19;3(1):e20260076. doi: 10.70962/jhi.20260076 (PMC13679715; doi:10.70962/jhi.20260076)
Supplement: Supplementary file 3 [file jhi_20260076_datas3.docx]

**Text S2.** Summarized Clinterms in Disease feature groups

Disease features:

1. Autoimmunity
2. Lymphoproliferation
3. Infections
   3.1. Viral infections
   3.2. Bacterial infections
   3.3. Fungal infections
   3.4. Parasite infections
4. Malignant neoplasm
5. Hypogammaglobulinemia
6. (Auto-)inflammation
7. CNS manifestation
8. Cardiovascular system abnormality
1. Autoimmunity

Alopecia

Alopecia areata

Alopecia totalis

anti-TPO antibodies

Anticardiolipin IgG antibody positivity

Antineutrophil antibodies

Antinuclear antibodies

Antiphospholipid antibody positive

Atrophic gastritis

Autoimmune cytopenia

Autoimmune enteropathy

Autoimmune hemolytic anemia

Autoimmune myocarditis

Autoimmune neutropenia

Autoimmune thrombocytopenia

Autoimmune thyroiditis

Autoimmunity

Biliary cholangitis

Celiac disease

Coombs Test Positive

Coombs-positive hemolytic anemia

Coombs-positive hemolytic anemia

Diabetes mellitus

Morphea

Multiple Sclerosis

Platelet antibody positive

Raynaud phenomenon

Rheumatoid factor positive
Rheumatoid arthritis

Small vessel vasculitis

Systemic lupus erythematosus

Type I diabetes mellitus

Vitiligo

2. Lymphoproliferation

Abnormal lymphocyte proliferation

B-cell lymphoma

Cervical lymphadenopathy

Chronic lymphatic leukemia

Enlarged mesenteric lymph node

Follicular hyperplasia

Follicular lymphoma

Generalized lymphadenopathy

Granulomatous-lymphocytic interstitial lung disease

Hepatomegaly

Hepatosplenomegaly

Intestinal lymphoid nodular hyperplasia

Large granular lymphocytic leukemia

Lymphadenitis

Lymphadenopathy

Lymphocytic colitis

Lymphoid hyperplasia

Lymphoid interstitial pneumonia

Lymphoproliferative disorder

Mediastinal lymphadenopathy

Multiple gastric polyps

Nodular regenerative hyperplasia of liver

Non-Hodgkin lymphoma

Splenomegaly

T-cell lymphoma

3. Infections

(Unusual) bacterial infection

(unusual) Epstein-Barr virus infection

(unusual) Fungal infection

(unusual) Giardiasis

(unusual) influenza infection

(unusual) Viral infection

Abscess

Bacterial mastitis

Bacterial meningitis

Bronchitis

Campylobacter infection

Cellulitis

Cholecystitis

Chronic furunculosis

Clostridium difficile colitis

Conjunctivitis

Cutaneous abscess

Cutaneous fungal infections

Cytomegalovirus colitis

Cytomegalovirus infection

Cytomegalovirus Infection Reactivation

Disseminated nontuberculous mycobacterial infection

Duodenal ulcer

Endocarditis

Enteritis

Epididymitis

Epiglottitis

Erysipelas

Esophageal candidiasis

Folliculitis

Furuncle

Gastrointestinal inflammation

Genital herpes

Gluteal abscess

Helicobacter pylori infection

Hepatitis B

Hepatitis C

Herpes labialis, recurrent

Herpes Simplex Virus Infection

HIV Infection

Human papilloma virus infection

Infection of the gastrointestinal tract

Invasive pulmonary aspergillosis

Liver abscess

Lung abscess

Lymph node abscess

Lymphadenitis

Mastoiditis

Measles

Meningitis

Molluscum contagiosum

Mucocutaneous candidiasis

Mumps

Mycobacterium Tuberculosis infection

Myocarditis

Necrotizing Tonsillitis

Neisseria meningitidis infection

Neonatal omphalitis

Neonatal sepsis

Norovirus infection

Onychomycosis

Oral candidiasis

Otitis media

Pancolitis

Parapharyngeal abscess

Paronychia

Parvovirus infection

Perianal Abscess

Periodontitis

Peritoneal abscess

Pharyngitis

Plantar warts

Pleural empyema

Pleural empyema due to Salmonella

Pneumocystis jiroveci pneumonia

Pneumonia

Progressive leukoencephalopathy

Progressive multifocal leukoencephalopathy

Prostatitis

Protozoan infection

Purulent rhinitis

Pyelonephritis

Recurrent bronchitis

Recurrent ear infections

Recurrent fungal infections

Recurrent Haemophilus influenzae infections

Recurrent infections

Recurrent lower respiratory tract infections

Recurrent mycobacterium avium complex infections

Recurrent opportunistic infections

Recurrent pharyngitis

Recurrent shingles

Recurrent streptococcus pneumoniae infections

Recurrent upper respiratory tract infection

Recurrent viral infections

Rhinitis

Rosacea

Rotavirus infection

RSV pneumonia

Rubella

Salmonella infection

Septicaemia

Severe SARS-CoV-2 infection

Sinopulmonary infections

Sinusitis

Skin infections

Streptococcal tonsillitis

Subglottic laryngitis

Suppurative Otitis Media

Syphilis

Tonsillitis

Tooth Abscess

Unusual gastrointestinal infection

Urinary tract infection

Vaginitis

Varicella zoster virus infection

Viral hepatitis

Viral meningitis

Vulvovaginal candidiasis

Warts

3.1 Viral infections

(unusual) Epstein-Barr virus infection

(unusual) influenza infection

(unusual) Viral infection

Cytomegalovirus colitis

Cytomegalovirus infection

Cytomegalovirus Infection Reactivation

Genital herpes

Hepatitis B

Hepatitis C

Herpes labialis, recurrent

Herpes Simplex Virus Infection

HIV Infection

Human papilloma virus infection

Measles

Molluscum contagiosum

Mumps

Myocarditis

Neisseria meningitidis infection

Norovirus infection

Parvovirus infection

Persistent CMV viremia

Plantar warts

Progressive leukoencephalopathy

Recurrent shingles

Recurrent viral infections

Rotavirus infection

RSV pneumonia

Rubella

Severe SARS-CoV-2 infection

Stomatitis

Subglottic laryngitis

Varicella zoster virus infection

Viral hepatitis

Viral meningitis

Warts

3.2 Bacterial infections

(Unusual) bacterial infection

Abscess

Bacterial mastitis

Bacterial meningitis

Campylobacter infection

Cellulitis

Chronic furunculosis

Clostridium difficile colitis

Cutaneous abscess

Disseminated nontuberculous mycobacterial infection

Duodenal ulcer

Endocarditis

Epiglottitis

Erysipelas

Folliculitis

Furuncle

Gluteal abscess

Helicobacter pylori infection

Liver abscess

Lung abscess

Mycobacterium Tuberculosis infection

Neonatal omphalitis

Onychomycosis

Parapharyngeal abscess

Paronychia

Perianal Abscess

Periodontitis

Pleural empyema

Pleural empyema due to Salmonella

Recurrent Haemophilus influenzae infections

Recurrent mycobacterium avium complex infections

Recurrent streptococcus pneumoniae infections

Salmonella infection

Streptococcal tonsillitis

Suppurative Otitis Media

Syphilis

3.3 Fungal infections

(unusual) Fungal infection

Cutaneous fungal infections

Esophageal candidiasis

Invasive pulmonary aspergillosis

Mucocutaneous candidiasis

Oral candidiasis

Pneumocystis jiroveci pneumonia

Recurrent fungal infections

Vulvovaginal candidiasis

3.4 Protozoan infections

(unusual) Giardiasis

Protozoan infection

4. Malignant neoplasm

B-cell lymphoma

Basal cell carcinoma

Breast carcinoma

cervical carcinoma

Chronic lymphatic leukemia

Follicular lymphoma

Large granular lymphocytic leukemia

Lung adenocarcinoma

Non-Hodgkin lymphoma

Renal neoplasm

Renal transitional cell carcinoma

Squamous cell carcinoma

Squamous cell carcinoma of the tongue

T-cell lymphoma

5. Hypogammaglobulinemia

Agammaglobulinemia

Decreased circulating IgG subclass level

Decreased IgA levels

Decreased IgE levels

Decreased IgG levels

Decreased IgM levels

Panhypogammaglobulinemia

Reduced IgG1 levels

Reduced IgG2 levels

Reduced IgG3 levels

Reduced IgG4 levels

6. (Auto-)inflammation

Angioedema

Aphthous oral ulcer

Arteritis

Atopic dermatitis

Behcet disease

Cirrhosis

Colitis

Crohn's disease

Eczema

Enteritis

Enteropathy

Eosinophilic esophagitis

erythema nodosum

Esophagitis

Gastritis

Genital ulcers

Hepatic fibrosis

Hepatic granulomatosis

Hepatic steatosis

Hepatitis

Hepatopathy

Immune dysregulation

Increased inflammatory response

Inflammation of the large intestine

Keratitis

Lichen Planus

Lymphocytic colitis

Lymphoid interstitial pneumonia

Microscopic colitis

Mucositis

Necrotizing cellulitis

Necrotizing enterocolitis

Necrotizing fasciitis

Neutrophilic infiltration of the skin

Optic neuritis

Optic papillitis

Oral ulcer

Orbital cellulitis

Pancreatitis

Parotitis

Pericarditis

Polymyalgia Rheumatica

Portal hypertension

Protein-losing enteropathy

Pruritus

Psoriasiform dermatitis

Psoriatic Arthritis

Pyoderma

Pyoderma gangrenosum

Retinitis

Sclerosing mesenteritis

Sjogren Syndrome

Small vessel vasculitis

Spondyloarthritis

Sterile abscess

Sterile sepsis

Stomatitis

ulcerative colitis

Urticaria

Vaginal mucosal ulceration

Vasculitis

7. CNS manifestations

Bacterial meningitis

Brachial plexus neuropathy

cerebral granulomatosis

Cerebral infarct

Cerebral vasculitis

Chronic CSF lymphocytosis

Delirium

Depressive disorder

Facial palsy

Headaches

Ischemic stroke

Meningitis

Migraine

Migraine with aura

Multiple Sclerosis

Myelitis

Non-infectious meningitis

Optic neuritis

optic papillitis

Paresthesia

Progressive leukoencephalopathy

Progressive multifocal leukoencephalopathy

Restless legs syndrome

Retinitis

Seizures

Subdural hemorrhage

Tremor

Viral meningitis

8. Cardiovascular system abnormality

Aortic regurgitation

Aortic valve stenosis

Arteritis

Autoimmune myocarditis

Congestive heart failure

Cor pulmonale

Coronary artery atherosclerosis

Deep venous thrombosis

Endocarditis

Hypotension

Myocardial infarction

Myocarditis

Pericarditis

Portal vein thrombosis

Pulmonary embolism

Raynaud phenomenon

varicose veins

Vasculitis

Venous thrombosis

Ventricular septal defect
